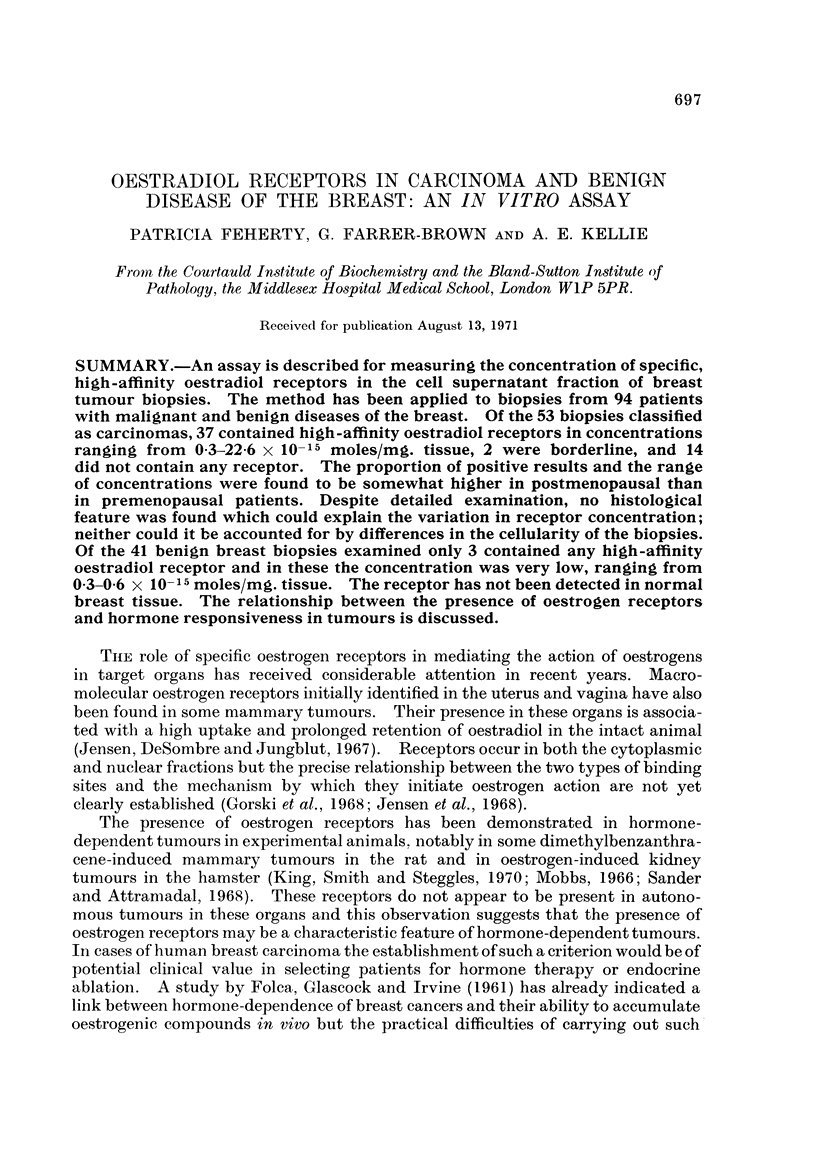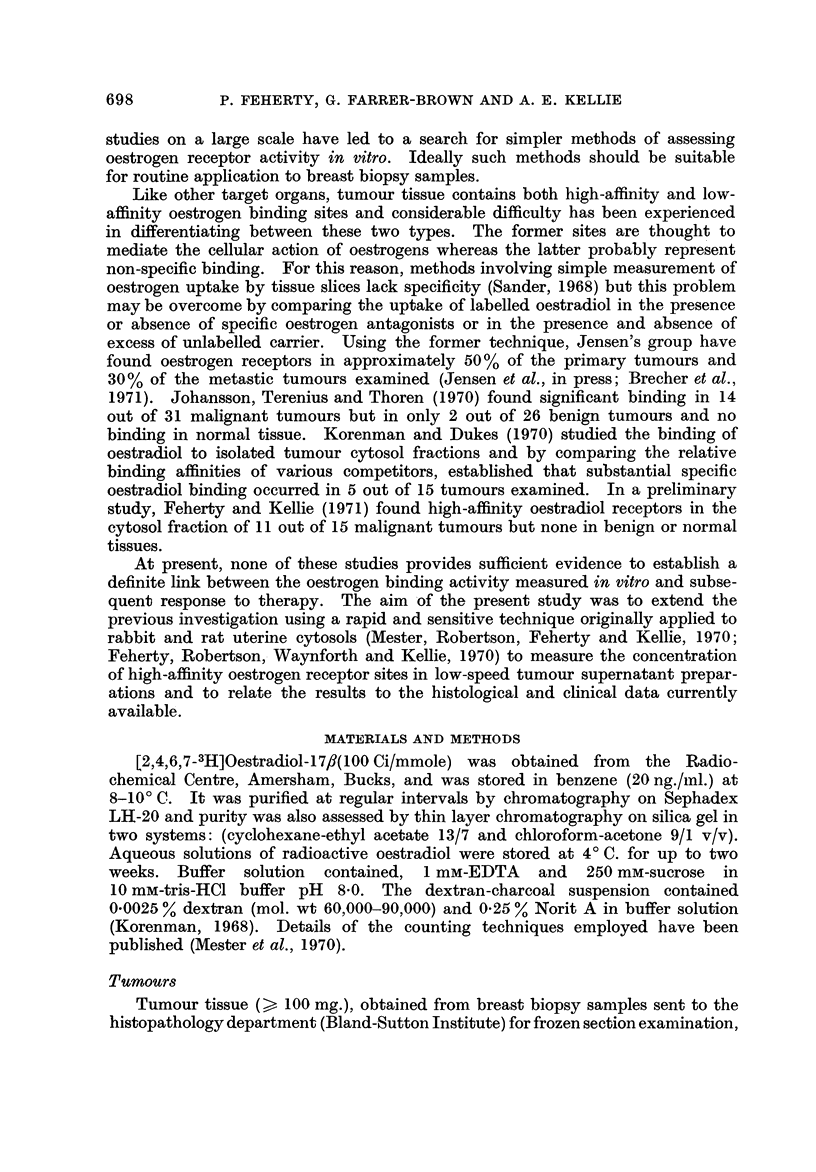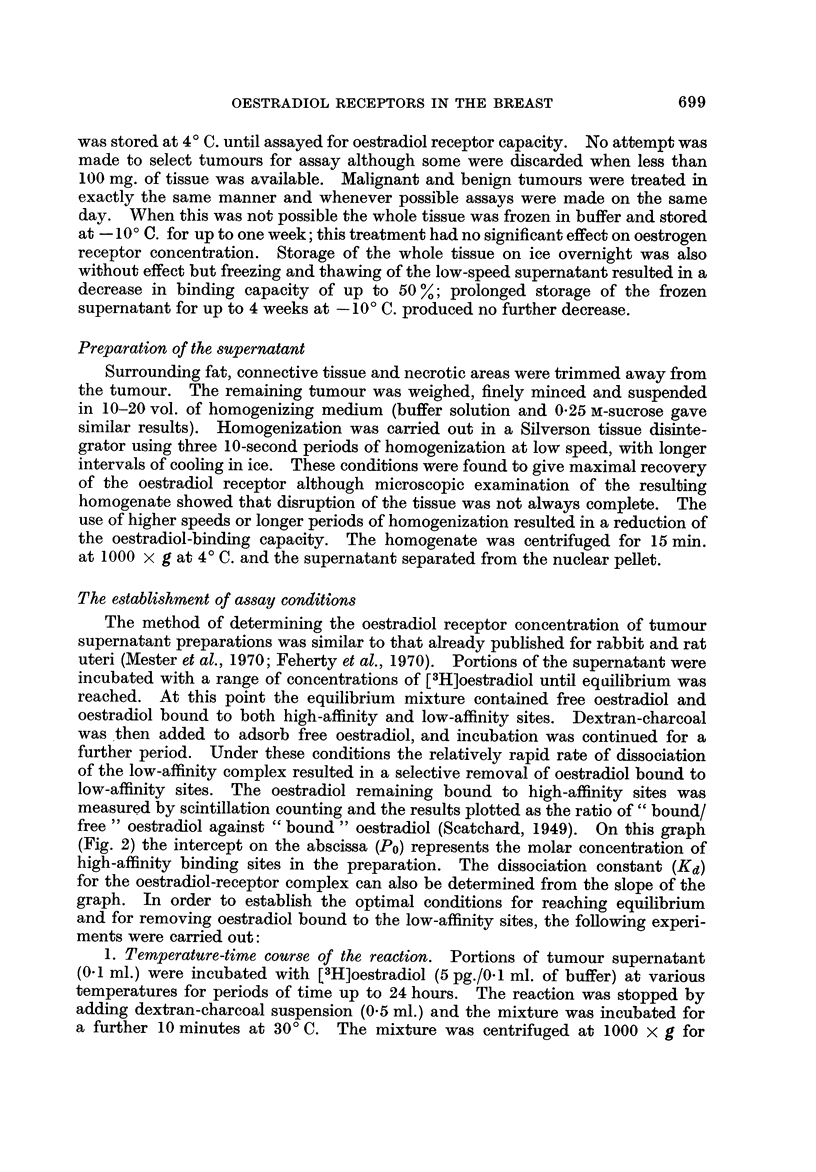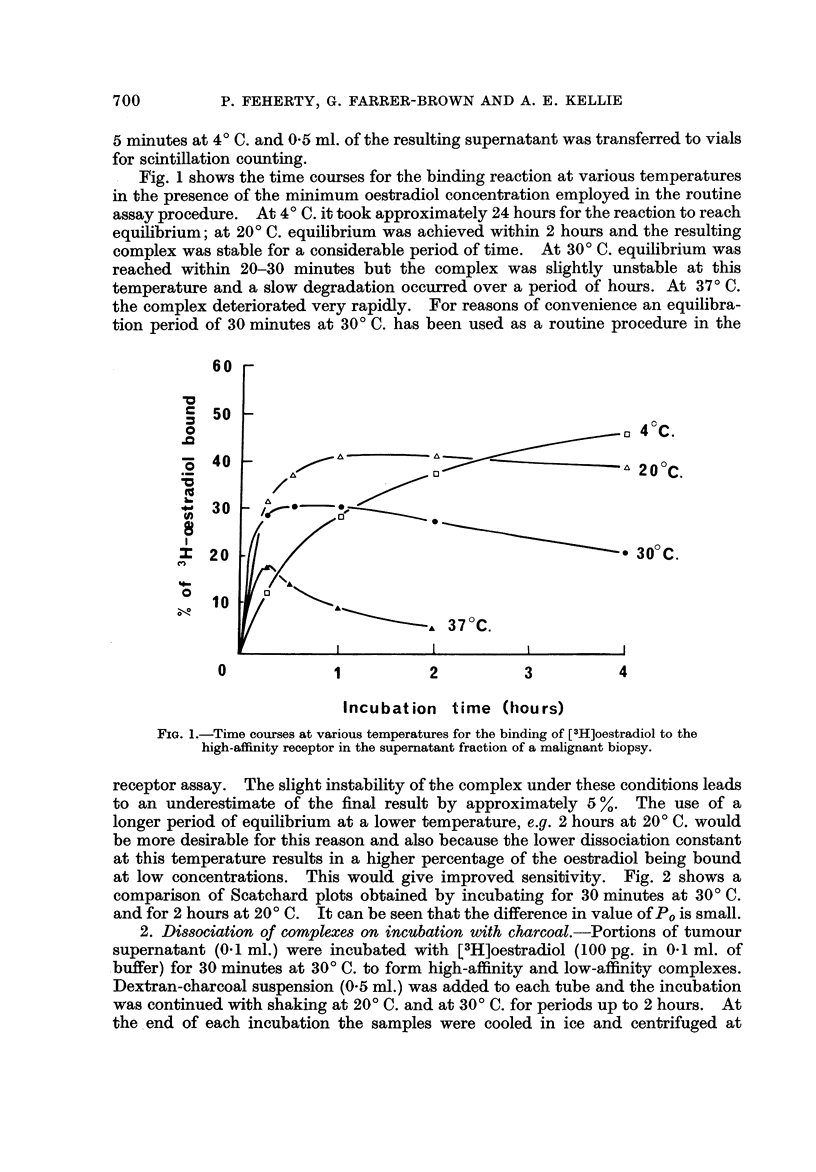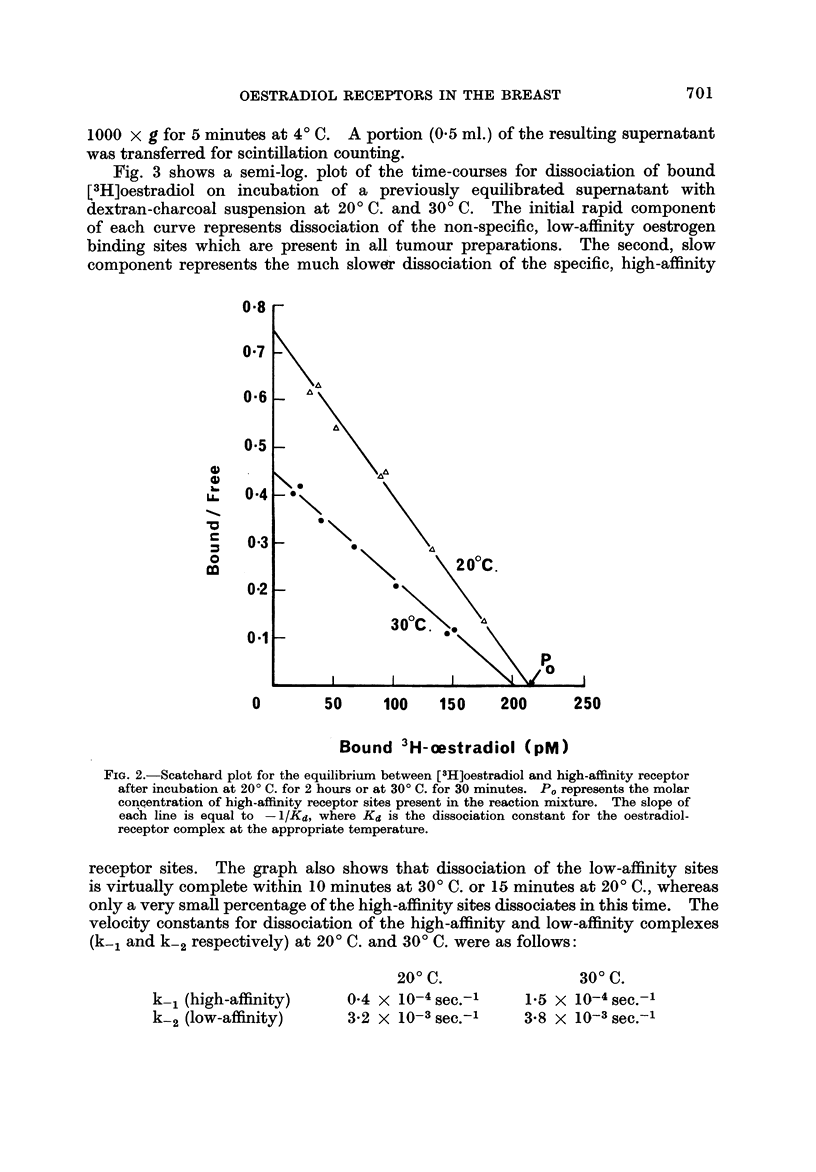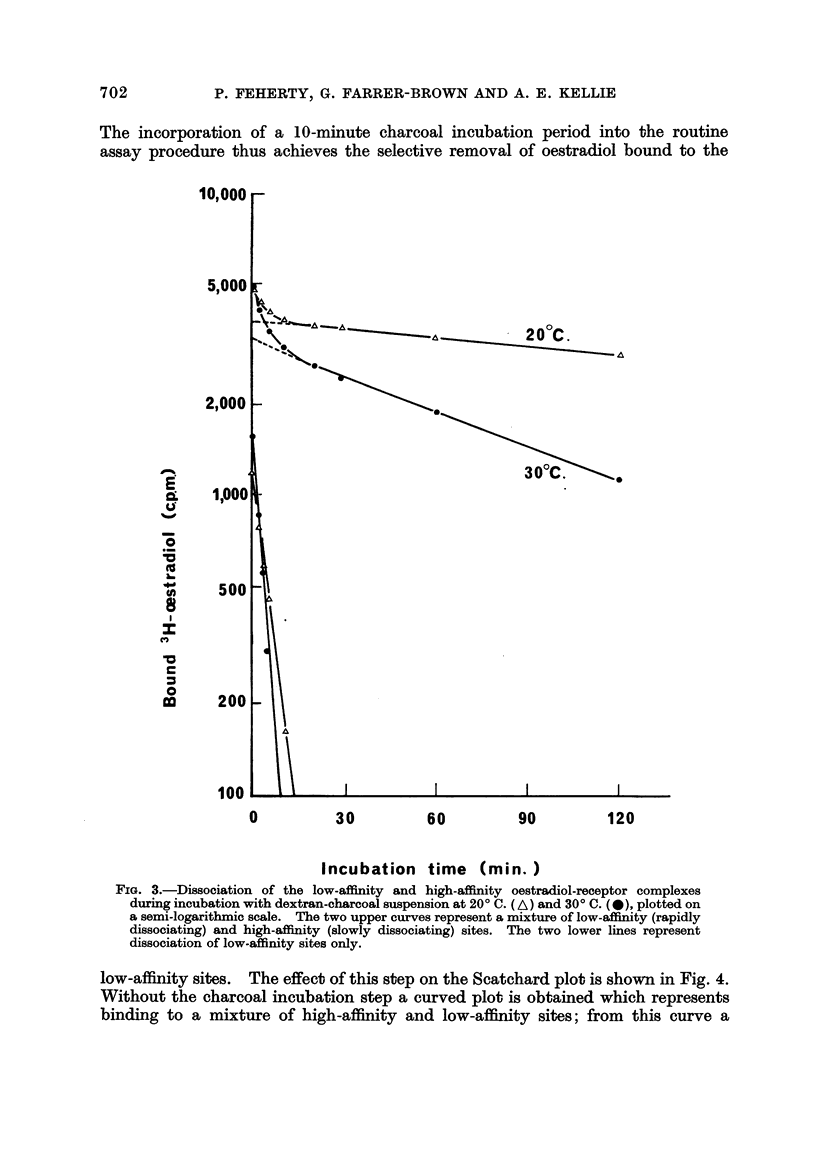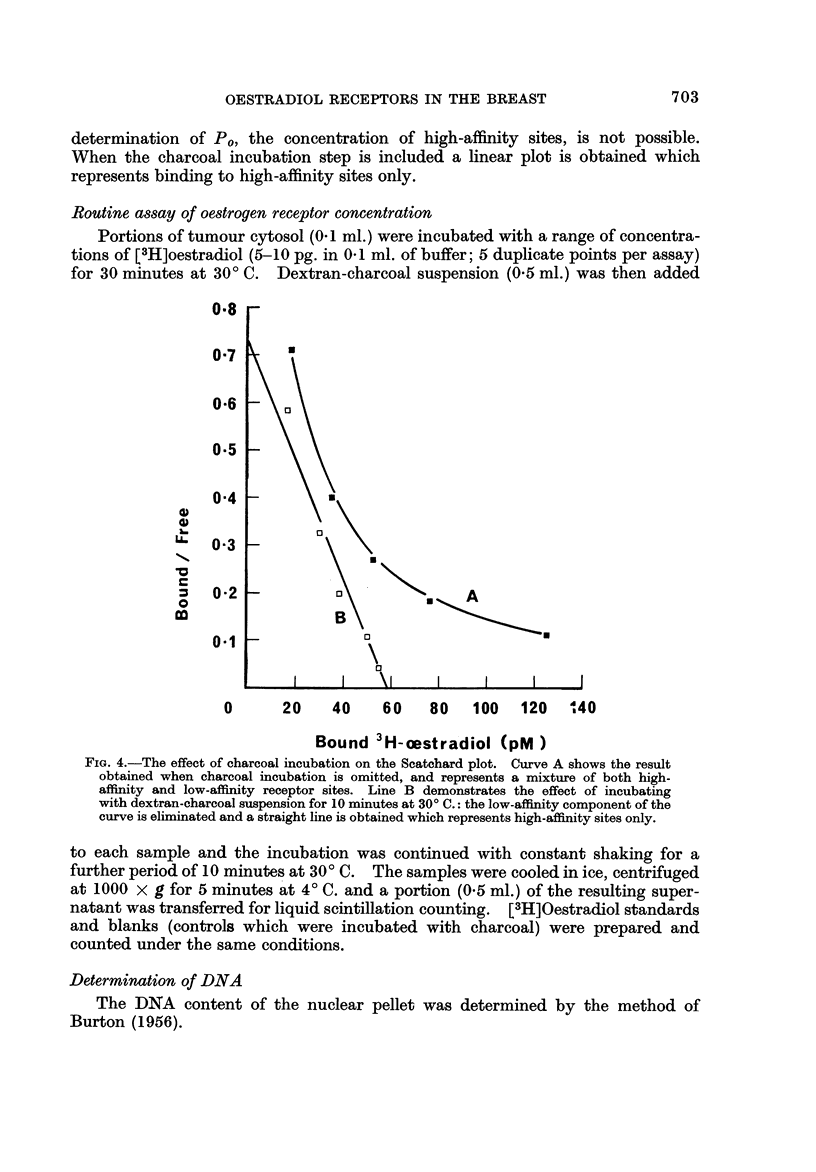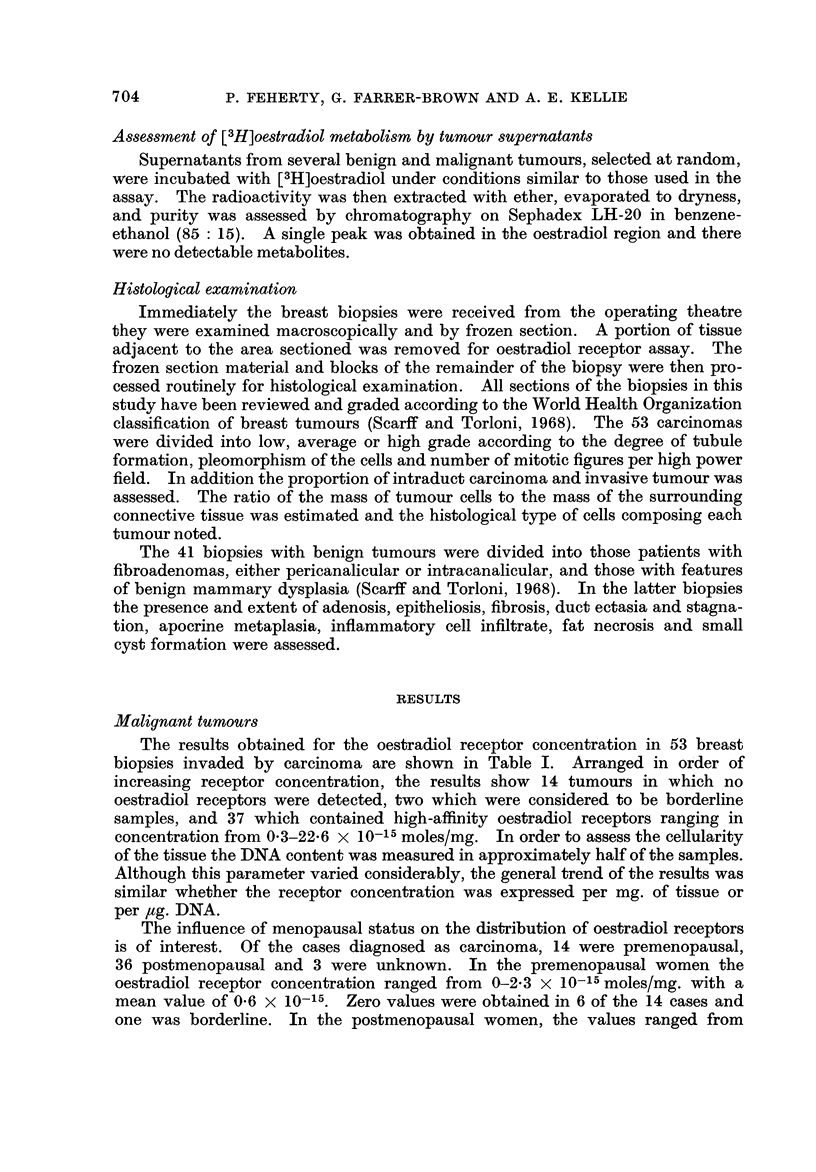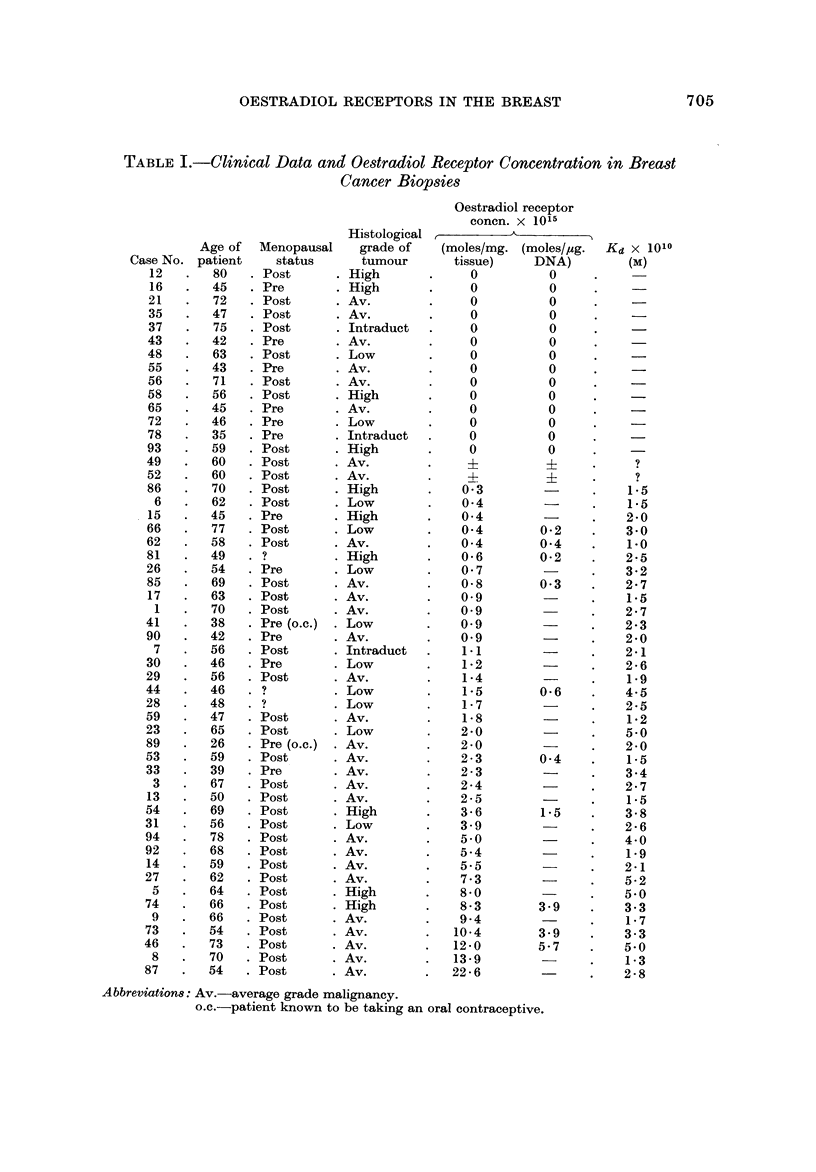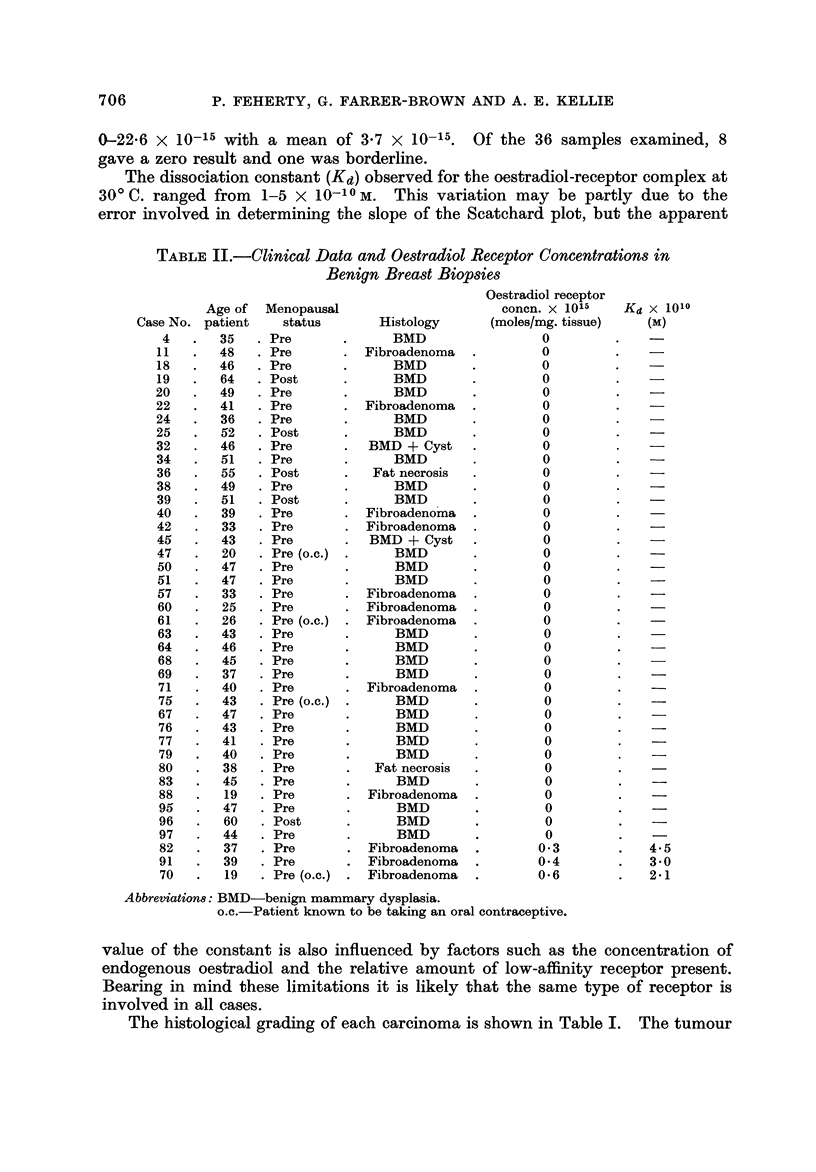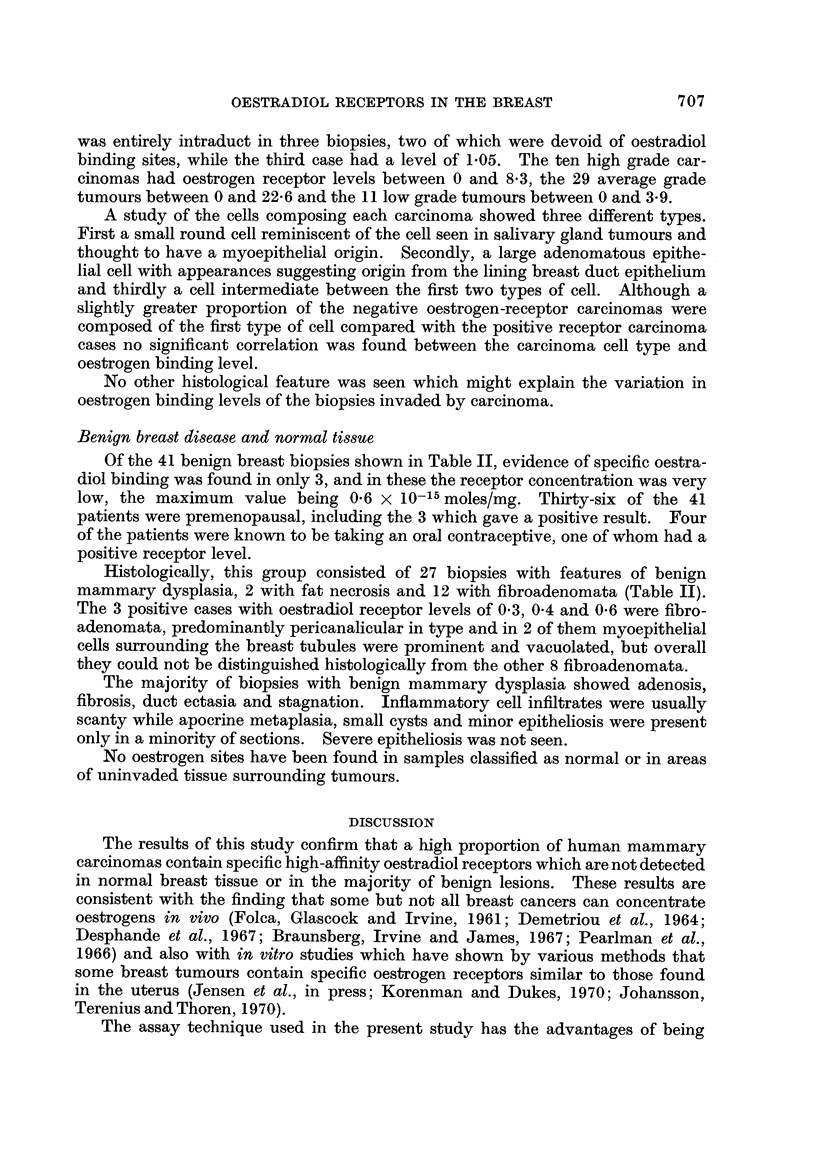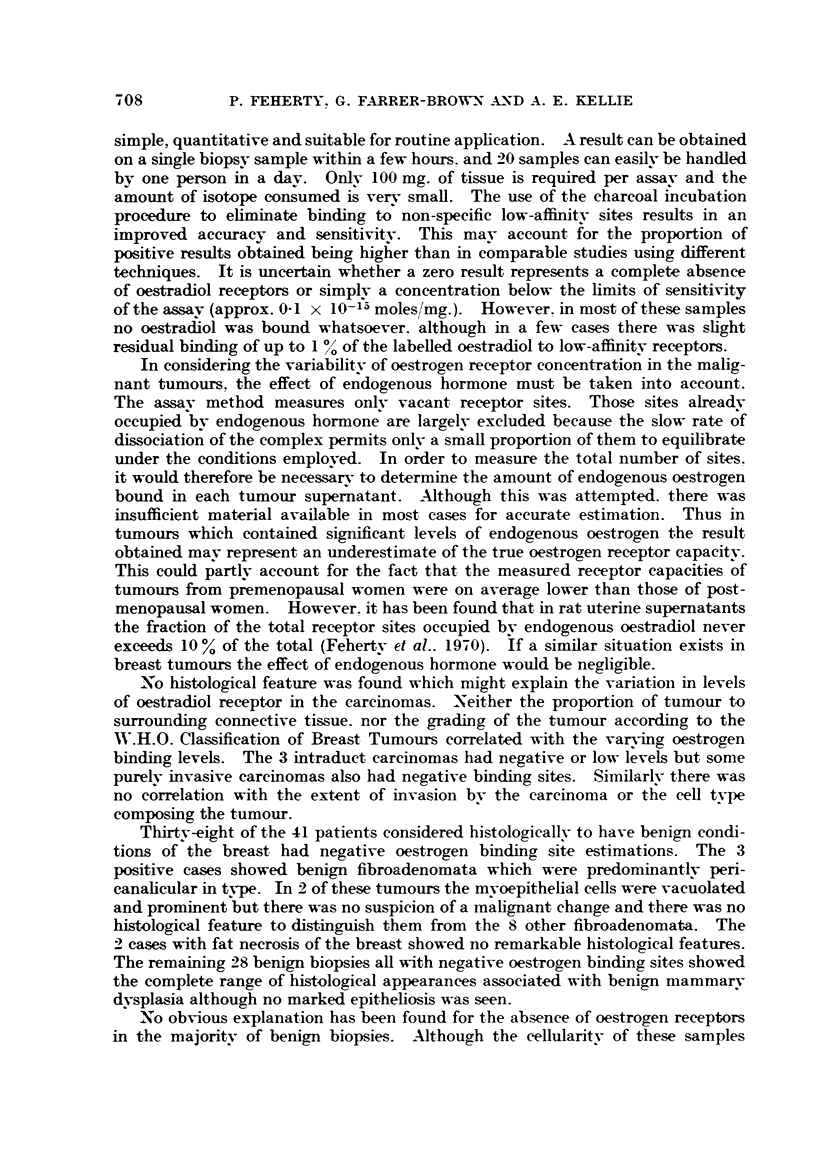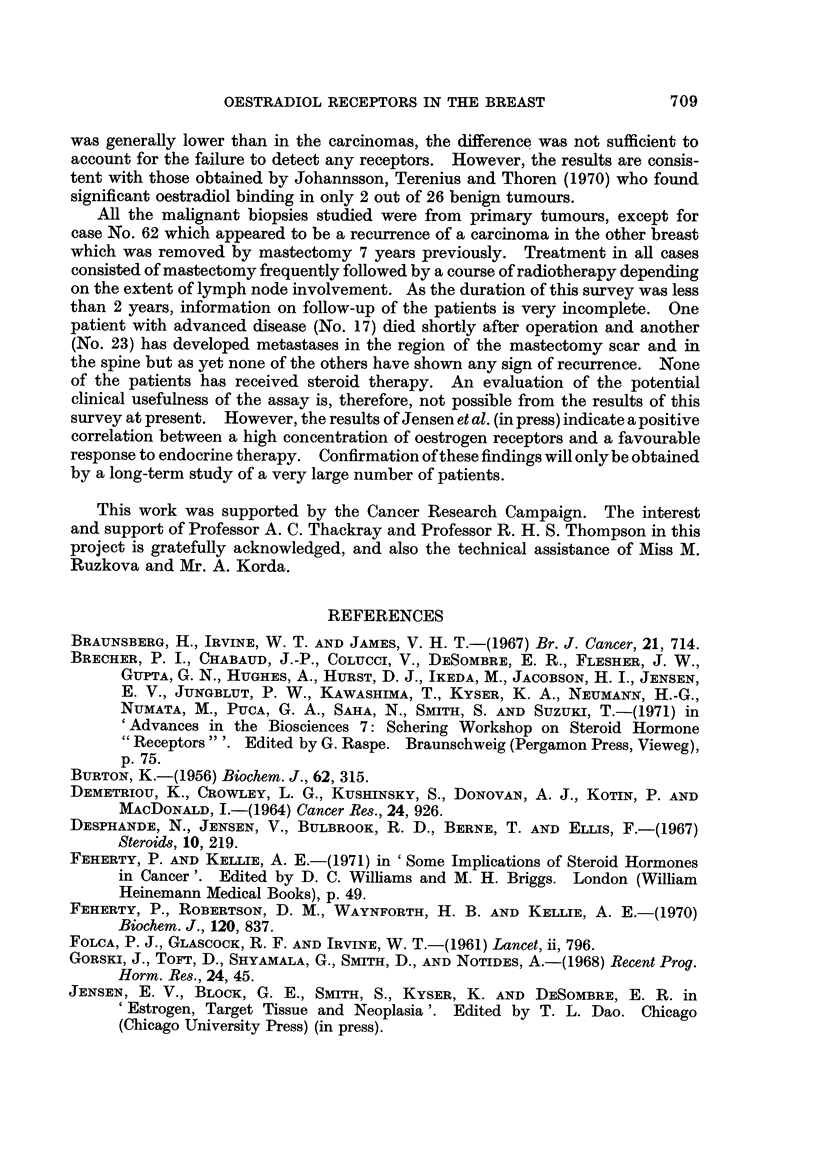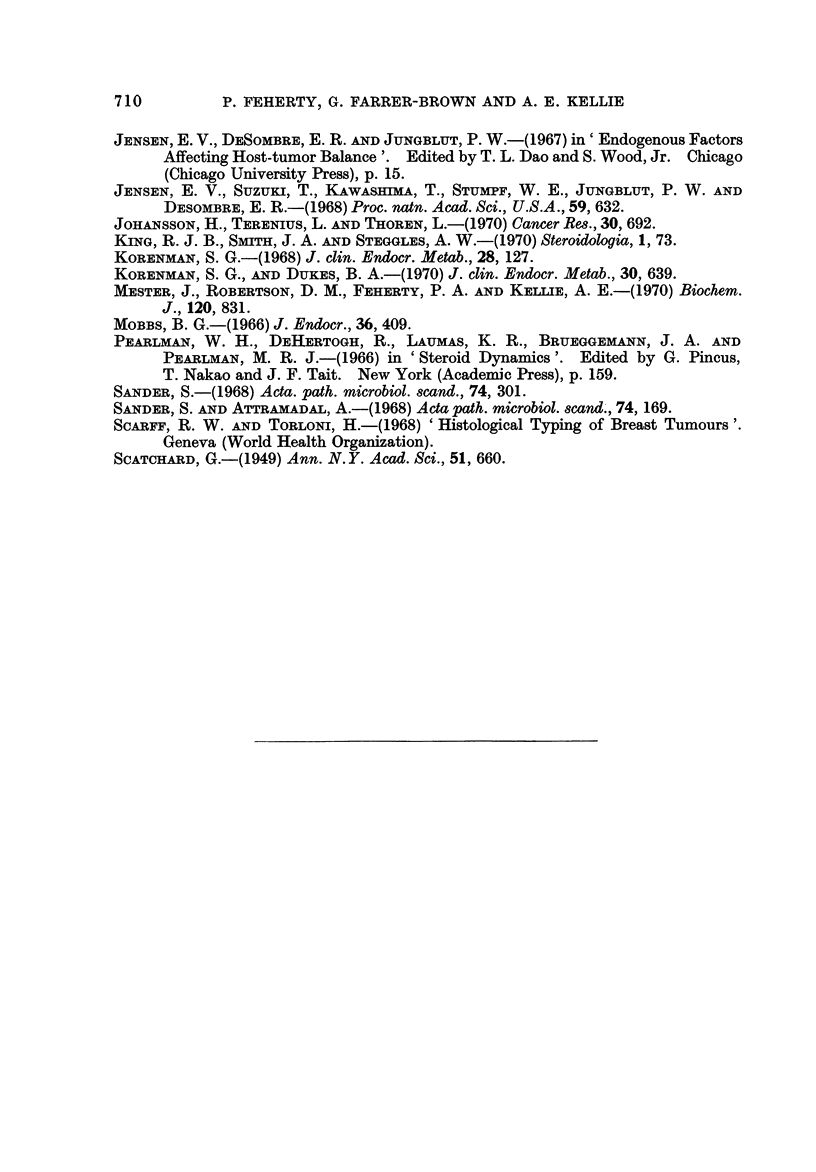# Oestradiol Receptors in Carcinoma and Benign Disease of the Breast: An In Vitro Assay

**DOI:** 10.1038/bjc.1971.85

**Published:** 1971-12

**Authors:** Patricia Feherty, G. Farrer-brown, A. E. Kellie

## Abstract

An assay is described for measuring the concentration of specific, high-affinity oestradiol receptors in the cell supernatant fraction of breast tumour biopsies. The method has been applied to biopsies from 94 patients with malignant and benign diseases of the breast. Of the 53 biopsies classified as carcinomas, 37 contained high-affinity oestradiol receptors in concentrations ranging from 0·3-22·6 × 10^-15^ moles/mg. tissue, 2 were borderline, and 14 did not contain any receptor. The proportion of positive results and the range of concentrations were found to be somewhat higher in postmenopausal than in premenopausal patients. Despite detailed examination, no histological feature was found which could explain the variation in receptor concentration; neither could it be accounted for by differences in the cellularity of the biopsies. Of the 41 benign breast biopsies examined only 3 contained any high-affinity oestradiol receptor and in these the concentration was very low, ranging from 0·3-0·6 × 10^-15^ moles/mg. tissue. The receptor has not been detected in normal breast tissue. The relationship between the presence of oestrogen receptors and hormone responsiveness in tumours is discussed.


### OESTRADIOL RECEPTORS IN CARCINOMA AND BENIGN DISEASE OF THE BREAST: AN *IN VITRO* ASSAY

PATRICIA FEHERTY, G. FARRER-BROWN AND A. E. KELLIE

*From the Courtauld Institute of Biochemistry and the Bland-Sutton Institute of Pathology, the Middlesex Hospital Medical School, London W1P 5PR.*

Received for publication August 13, 1971

**SUMMARY.—An assay is described for measuring the concentration of specific, high-affinity oestradiol receptors in the cell supernatant fraction of breast tumour biopsies. The method has been applied to biopsies from 94 patients with malignant and benign diseases of the breast. Of the 53 biopsies classified as carcinomas, 37 contained high-affinity oestradiol receptors in concentrations ranging from 0·3–22·6 $\times$ $10^{-15}$ moles/mg. tissue, 2 were borderline, and 14 did not contain any receptor. The proportion of positive results and the range of concentrations were found to be somewhat higher in postmenopausal than in premenopausal patients. Despite detailed examination, no histological feature was found which could explain the variation in receptor concentration; neither could it be accounted for by differences in the cellularity of the biopsies. Of the 41 benign breast biopsies examined only 3 contained any high-affinity oestradiol receptor and in these the concentration was very low, ranging from 0·3–0·6 $\times$ $10^{-15}$ moles/mg. tissue. The receptor has not been detected in normal breast tissue. The relationship between the presence of oestrogen receptors and hormone responsiveness in tumours is discussed.**

THE role of specific oestrogen receptors in mediating the action of oestrogens in target organs has received considerable attention in recent years. Macromolecular oestrogen receptors initially identified in the uterus and vagina have also been found in some mammary tumours. Their presence in these organs is associated with a high uptake and prolonged retention of oestradiol in the intact animal (Jensen, DeSombre and Jungblut, 1967). Receptors occur in both the cytoplasmic and nuclear fractions but the precise relationship between the two types of binding sites and the mechanism by which they initiate oestrogen action are not yet clearly established (Gorski *et al.*, 1968; Jensen *et al.*, 1968).

The presence of oestrogen receptors has been demonstrated in hormone-dependent tumours in experimental animals, notably in some dimethylbenzanthracene-induced mammary tumours in the rat and in oestrogen-induced kidney tumours in the hamster (King, Smith and Steggles, 1970; Mobbs, 1966; Sander and Attramadal, 1968). These receptors do not appear to be present in autonomous tumours in these organs and this observation suggests that the presence of oestrogen receptors may be a characteristic feature of hormone-dependent tumours. In cases of human breast carcinoma the establishment of such a criterion would be of potential clinical value in selecting patients for hormone therapy or endocrine ablation. A study by Folca, Glascock and Irvine (1961) has already indicated a link between hormone-dependence of breast cancers and their ability to accumulate oestrogenic compounds *in vivo* but the practical difficulties of carrying out such studies on a large scale have led to a search for simpler methods of assessing oestrogen receptor activity *in vitro*. Ideally such methods should be suitable for routine application to breast biopsy samples.

Like other target organs, tumour tissue contains both high-affinity and low-affinity oestrogen binding sites and considerable difficulty has been experienced in differentiating between these two types. The former sites are thought to mediate the cellular action of oestrogens whereas the latter probably represent non-specific binding. For this reason, methods involving simple measurement of oestrogen uptake by tissue slices lack specificity (Sander, 1968) but this problem may be overcome by comparing the uptake of labelled oestradiol in the presence or absence of specific oestrogen antagonists or in the presence and absence of excess of unlabelled carrier. Using the former technique, Jensen's group have found oestrogen receptors in approximately 50% of the primary tumours and 30% of the metastic tumours examined (Jensen *et al.*, in press; Brecher *et al.*, 1971). Johansson, Terenius and Thoren (1970) found significant binding in 14 out of 31 malignant tumours but in only 2 out of 26 benign tumours and no binding in normal tissue. Korenman and Dukes (1970) studied the binding of oestradiol to isolated tumour cytosol fractions and by comparing the relative binding affinities of various competitors, established that substantial specific oestradiol binding occurred in 5 out of 15 tumours examined. In a preliminary study, Feherty and Kellie (1971) found high-affinity oestradiol receptors in the cytosol fraction of 11 out of 15 malignant tumours but none in benign or normal tissues.

At present, none of these studies provides sufficient evidence to establish a definite link between the oestrogen binding activity measured *in vitro* and subsequent response to therapy. The aim of the present study was to extend the previous investigation using a rapid and sensitive technique originally applied to rabbit and rat uterine cytosols (Mester, Robertson, Feherty and Kellie, 1970; Feherty, Robertson, Waynforth and Kellie, 1970) to measure the concentration of high-affinity oestrogen receptor sites in low-speed tumour supernatant preparations and to relate the results to the histological and clinical data currently available.

### MATERIALS AND METHODS

[2,4,6,7-$^3$H]Oestradiol-17$\beta$(100 Ci/mmole) was obtained from the Radiochemical Centre, Amersham, Bucks, and was stored in benzene (20 ng./ml.) at 8–10° C. It was purified at regular intervals by chromatography on Sephadex LH-20 and purity was also assessed by thin layer chromatography on silica gel in two systems: (cyclohexane-ethyl acetate 13/7 and chloroform-acetone 9/1 v/v). Aqueous solutions of radioactive oestradiol were stored at 4° C. for up to two weeks. Buffer solution contained, 1 mM-EDTA and 250 mM-sucrose in 10 mM-tris-HCl buffer pH 8·0. The dextran-charcoal suspension contained 0·0025% dextran (mol. wt 60,000–90,000) and 0·25% Norit A in buffer solution (Korenman, 1968). Details of the counting techniques employed have been published (Mester *et al.*, 1970).

### *Tumours*

Tumour tissue ($\geqslant$ 100 mg.), obtained from breast biopsy samples sent to the histopathology department (Bland-Sutton Institute) for frozen section examination, was stored at 4° C. until assayed for oestradiol receptor capacity. No attempt was made to select tumours for assay although some were discarded when less than 100 mg. of tissue was available. Malignant and benign tumours were treated in exactly the same manner and whenever possible assays were made on the same day. When this was not possible the whole tissue was frozen in buffer and stored at −10° C. for up to one week; this treatment had no significant effect on oestrogen receptor concentration. Storage of the whole tissue on ice overnight was also without effect but freezing and thawing of the low-speed supernatant resulted in a decrease in binding capacity of up to 50%; prolonged storage of the frozen supernatant for up to 4 weeks at −10° C. produced no further decrease.

*Preparation of the supernatant*

Surrounding fat, connective tissue and necrotic areas were trimmed away from the tumour. The remaining tumour was weighed, finely minced and suspended in 10–20 vol. of homogenizing medium (buffer solution and 0·25 M-sucrose gave similar results). Homogenization was carried out in a Silverson tissue disintegrator using three 10-second periods of homogenization at low speed, with longer intervals of cooling in ice. These conditions were found to give maximal recovery of the oestradiol receptor although microscopic examination of the resulting homogenate showed that disruption of the tissue was not always complete. The use of higher speeds or longer periods of homogenization resulted in a reduction of the oestradiol-binding capacity. The homogenate was centrifuged for 15 min. at 1000 × $g$ at 4° C. and the supernatant separated from the nuclear pellet.

*The establishment of assay conditions*

The method of determining the oestradiol receptor concentration of tumour supernatant preparations was similar to that already published for rabbit and rat uteri (Mester *et al.*, 1970; Feherty *et al.*, 1970). Portions of the supernatant were incubated with a range of concentrations of [$^3$H]oestradiol until equilibrium was reached. At this point the equilibrium mixture contained free oestradiol and oestradiol bound to both high-affinity and low-affinity sites. Dextran-charcoal was then added to adsorb free oestradiol, and incubation was continued for a further period. Under these conditions the relatively rapid rate of dissociation of the low-affinity complex resulted in a selective removal of oestradiol bound to low-affinity sites. The oestradiol remaining bound to high-affinity sites was measured by scintillation counting and the results plotted as the ratio of "bound/free" oestradiol against "bound" oestradiol (Scatchard, 1949). On this graph (Fig. 2) the intercept on the abscissa ($P_0$) represents the molar concentration of high-affinity binding sites in the preparation. The dissociation constant ($K_d$) for the oestradiol-receptor complex can also be determined from the slope of the graph. In order to establish the optimal conditions for reaching equilibrium and for removing oestradiol bound to the low-affinity sites, the following experiments were carried out:

1. *Temperature-time course of the reaction.* Portions of tumour supernatant (0·1 ml.) were incubated with [$^3$H]oestradiol (5 pg./0·1 ml. of buffer) at various temperatures for periods of time up to 24 hours. The reaction was stopped by adding dextran-charcoal suspension (0·5 ml.) and the mixture was incubated for a further 10 minutes at 30° C. The mixture was centrifuged at 1000 × $g$ for

5 minutes at 4° C. and 0·5 ml. of the resulting supernatant was transferred to vials for scintillation counting.

Fig. 1 shows the time courses for the binding reaction at various temperatures in the presence of the minimum oestradiol concentration employed in the routine assay procedure. At 4° C. it took approximately 24 hours for the reaction to reach equilibrium; at 20° C. equilibrium was achieved within 2 hours and the resulting complex was stable for a considerable period of time. At 30° C. equilibrium was reached within 20–30 minutes but the complex was slightly unstable at this temperature and a slow degradation occurred over a period of hours. At 37° C. the complex deteriorated very rapidly. For reasons of convenience an equilibration period of 30 minutes at 30° C. has been used as a routine procedure in the

FIG. 1.—Time courses at various temperatures for the binding of [$^3$H]oestradiol to the high-affinity receptor in the supernatant fraction of a malignant biopsy.

receptor assay. The slight instability of the complex under these conditions leads to an underestimate of the final result by approximately 5 %. The use of a longer period of equilibrium at a lower temperature, *e.g.* 2 hours at 20° C. would be more desirable for this reason and also because the lower dissociation constant at this temperature results in a higher percentage of the oestradiol being bound at low concentrations. This would give improved sensitivity. Fig. 2 shows a comparison of Scatchard plots obtained by incubating for 30 minutes at 30° C. and for 2 hours at 20° C. It can be seen that the difference in value of $P_0$ is small.

2. *Dissociation of complexes on incubation with charcoal.*—Portions of tumour supernatant (0·1 ml.) were incubated with [$^3$H]oestradiol (100 pg. in 0·1 ml. of buffer) for 30 minutes at 30° C. to form high-affinity and low-affinity complexes. Dextran-charcoal suspension (0·5 ml.) was added to each tube and the incubation was continued with shaking at 20° C. and at 30° C. for periods up to 2 hours. At the end of each incubation the samples were cooled in ice and centrifuged at

1000 × *g* for 5 minutes at 4° C. A portion (0·5 ml.) of the resulting supernatant was transferred for scintillation counting.

Fig. 3 shows a semi-log. plot of the time-courses for dissociation of bound [$^3$H]oestradiol on incubation of a previously equilibrated supernatant with dextran-charcoal suspension at 20° C. and 30° C. The initial rapid component of each curve represents dissociation of the non-specific, low-affinity oestrogen binding sites which are present in all tumour preparations. The second, slow component represents the much slower dissociation of the specific, high-affinity

FIG. 2.—Scatchard plot for the equilibrium between [$^3$H]oestradiol and high-affinity receptor after incubation at 20° C. for 2 hours or at 30° C. for 30 minutes. $P_0$ represents the molar concentration of high-affinity receptor sites present in the reaction mixture. The slope of each line is equal to $-1/K_d$, where $K_d$ is the dissociation constant for the oestradiol-receptor complex at the appropriate temperature.

receptor sites. The graph also shows that dissociation of the low-affinity sites is virtually complete within 10 minutes at 30° C. or 15 minutes at 20° C., whereas only a very small percentage of the high-affinity sites dissociates in this time. The velocity constants for dissociation of the high-affinity and low-affinity complexes ($k_{-1}$ and $k_{-2}$ respectively) at 20° C. and 30° C. were as follows:

| | 20° C. | 30° C. |
|---|---|---|
| $k_{-1}$ (high-affinity) | $0·4 \times 10^{-4}$ sec.$^{-1}$ | $1·5 \times 10^{-4}$ sec.$^{-1}$ |
| $k_{-2}$ (low-affinity) | $3·2 \times 10^{-3}$ sec.$^{-1}$ | $3·8 \times 10^{-3}$ sec.$^{-1}$ |

The incorporation of a 10-minute charcoal incubation period into the routine assay procedure thus achieves the selective removal of oestradiol bound to the

FIG. 3.—Dissociation of the low-affinity and high-affinity oestradiol-receptor complexes during incubation with dextran-charcoal suspension at 20° C. (△) and 30° C. (●), plotted on a semi-logarithmic scale. The two upper curves represent a mixture of low-affinity (rapidly dissociating) and high-affinity (slowly dissociating) sites. The two lower lines represent dissociation of low-affinity sites only.

low-affinity sites. The effect of this step on the Scatchard plot is shown in Fig. 4. Without the charcoal incubation step a curved plot is obtained which represents binding to a mixture of high-affinity and low-affinity sites; from this curve a determination of $P_0$, the concentration of high-affinity sites, is not possible. When the charcoal incubation step is included a linear plot is obtained which represents binding to high-affinity sites only.

*Routine assay of oestrogen receptor concentration*

Portions of tumour cytosol (0·1 ml.) were incubated with a range of concentrations of [$^3$H]oestradiol (5–10 pg. in 0·1 ml. of buffer; 5 duplicate points per assay) for 30 minutes at 30° C. Dextran-charcoal suspension (0·5 ml.) was then added

FIG. 4.—The effect of charcoal incubation on the Scatchard plot. Curve A shows the result obtained when charcoal incubation is omitted, and represents a mixture of both high-affinity and low-affinity receptor sites. Line B demonstrates the effect of incubating with dextran-charcoal suspension for 10 minutes at 30° C.: the low-affinity component of the curve is eliminated and a straight line is obtained which represents high-affinity sites only.

to each sample and the incubation was continued with constant shaking for a further period of 10 minutes at 30° C. The samples were cooled in ice, centrifuged at 1000 × $g$ for 5 minutes at 4° C. and a portion (0·5 ml.) of the resulting supernatant was transferred for liquid scintillation counting. [$^3$H]Oestradiol standards and blanks (controls which were incubated with charcoal) were prepared and counted under the same conditions.

*Determination of DNA*

The DNA content of the nuclear pellet was determined by the method of Burton (1956).

*Assessment of [$^3$H]oestradiol metabolism by tumour supernatants*

Supernatants from several benign and malignant tumours, selected at random, were incubated with [$^3$H]oestradiol under conditions similar to those used in the assay. The radioactivity was then extracted with ether, evaporated to dryness, and purity was assessed by chromatography on Sephadex LH-20 in benzene-ethanol (85 : 15). A single peak was obtained in the oestradiol region and there were no detectable metabolites.

*Histological examination*

Immediately the breast biopsies were received from the operating theatre they were examined macroscopically and by frozen section. A portion of tissue adjacent to the area sectioned was removed for oestradiol receptor assay. The frozen section material and blocks of the remainder of the biopsy were then processed routinely for histological examination. All sections of the biopsies in this study have been reviewed and graded according to the World Health Organization classification of breast tumours (Scarff and Torloni, 1968). The 53 carcinomas were divided into low, average or high grade according to the degree of tubule formation, pleomorphism of the cells and number of mitotic figures per high power field. In addition the proportion of intraduct carcinoma and invasive tumour was assessed. The ratio of the mass of tumour cells to the mass of the surrounding connective tissue was estimated and the histological type of cells composing each tumour noted.

The 41 biopsies with benign tumours were divided into those patients with fibroadenomas, either pericanalicular or intracanalicular, and those with features of benign mammary dysplasia (Scarff and Torloni, 1968). In the latter biopsies the presence and extent of adenosis, epitheliosis, fibrosis, duct ectasia and stagnation, apocrine metaplasia, inflammatory cell infiltrate, fat necrosis and small cyst formation were assessed.

### RESULTS

*Malignant tumours*

The results obtained for the oestradiol receptor concentration in 53 breast biopsies invaded by carcinoma are shown in Table I. Arranged in order of increasing receptor concentration, the results show 14 tumours in which no oestradiol receptors were detected, two which were considered to be borderline samples, and 37 which contained high-affinity oestradiol receptors ranging in concentration from 0·3–22·6 $\times$ $10^{-15}$ moles/mg. In order to assess the cellularity of the tissue the DNA content was measured in approximately half of the samples. Although this parameter varied considerably, the general trend of the results was similar whether the receptor concentration was expressed per mg. of tissue or per $\mu$g. DNA.

The influence of menopausal status on the distribution of oestradiol receptors is of interest. Of the cases diagnosed as carcinoma, 14 were premenopausal, 36 postmenopausal and 3 were unknown. In the premenopausal women the oestradiol receptor concentration ranged from 0–2·3 $\times$ $10^{-15}$ moles/mg. with a mean value of 0·6 $\times$ $10^{-15}$. Zero values were obtained in 6 of the 14 cases and one was borderline. In the postmenopausal women, the values ranged from

TABLE I.—*Clinical Data and Oestradiol Receptor Concentration in Breast Cancer Biopsies*

| Case No. | Age of patient | Menopausal status | Histological grade of tumour | Oestradiol receptor concn. × $10^{15}$ (moles/mg. tissue) | Oestradiol receptor concn. × $10^{15}$ (moles/μg. DNA) | $K_d \times 10^{10}$ (M) |
|---|---|---|---|---|---|---|
| 12 | 80 | Post | High | 0 | 0 | — |
| 16 | 45 | Pre | High | 0 | 0 | — |
| 21 | 72 | Post | Av. | 0 | 0 | — |
| 35 | 47 | Post | Av. | 0 | 0 | — |
| 37 | 75 | Post | Intraduct | 0 | 0 | — |
| 43 | 42 | Pre | Av. | 0 | 0 | — |
| 48 | 63 | Post | Low | 0 | 0 | — |
| 55 | 43 | Pre | Av. | 0 | 0 | — |
| 56 | 71 | Post | Av. | 0 | 0 | — |
| 58 | 56 | Post | High | 0 | 0 | — |
| 65 | 45 | Pre | Av. | 0 | 0 | — |
| 72 | 46 | Pre | Low | 0 | 0 | — |
| 78 | 35 | Pre | Intraduct | 0 | 0 | — |
| 93 | 59 | Post | High | 0 | 0 | — |
| 49 | 60 | Post | Av. | ± | ± | ? |
| 52 | 60 | Post | Av. | ± | ± | ? |
| 86 | 70 | Post | High | 0·3 | — | 1·5 |
| 6 | 62 | Post | Low | 0·4 | — | 1·5 |
| 15 | 45 | Pre | High | 0·4 | — | 2·0 |
| 66 | 77 | Post | Low | 0·4 | 0·2 | 3·0 |
| 62 | 58 | Post | Av. | 0·4 | 0·4 | 1·0 |
| 81 | 49 | ? | High | 0·6 | 0·2 | 2·5 |
| 26 | 54 | Pre | Low | 0·7 | — | 3·2 |
| 85 | 69 | Post | Av. | 0·8 | 0·3 | 2·7 |
| 17 | 63 | Post | Av. | 0·9 | — | 1·5 |
| 1 | 70 | Post | Av. | 0·9 | — | 2·7 |
| 41 | 38 | Pre (o.c.) | Low | 0·9 | — | 2·3 |
| 90 | 42 | Pre | Av. | 0·9 | — | 2·0 |
| 7 | 56 | Post | Intraduct | 1·1 | — | 2·1 |
| 30 | 46 | Pre | Low | 1·2 | — | 2·6 |
| 29 | 56 | Post | Av. | 1·4 | — | 1·9 |
| 44 | 46 | ? | Low | 1·5 | 0·6 | 4·5 |
| 28 | 48 | ? | Low | 1·7 | — | 2·5 |
| 59 | 47 | Post | Av. | 1·8 | — | 1·2 |
| 23 | 65 | Post | Low | 2·0 | — | 5·0 |
| 89 | 26 | Pre (o.c.) | Av. | 2·0 | — | 2·0 |
| 53 | 59 | Post | Av. | 2·3 | 0·4 | 1·5 |
| 33 | 39 | Pre | Av. | 2·3 | — | 3·4 |
| 3 | 67 | Post | Av. | 2·4 | — | 2·7 |
| 13 | 50 | Post | Av. | 2·5 | — | 1·5 |
| 54 | 69 | Post | High | 3·6 | 1·5 | 3·8 |
| 31 | 56 | Post | Low | 3·9 | — | 2·6 |
| 94 | 78 | Post | Av. | 5·0 | — | 4·0 |
| 92 | 68 | Post | Av. | 5·4 | — | 1·9 |
| 14 | 59 | Post | Av. | 5·5 | — | 2·1 |
| 27 | 62 | Post | Av. | 7·3 | — | 5·2 |
| 5 | 64 | Post | High | 8·0 | — | 5·0 |
| 74 | 66 | Post | High | 8·3 | 3·9 | 3·3 |
| 9 | 66 | Post | Av. | 9·4 | — | 1·7 |
| 73 | 54 | Post | Av. | 10·4 | 3·9 | 3·3 |
| 46 | 73 | Post | Av. | 12·0 | 5·7 | 5·0 |
| 8 | 70 | Post | Av. | 13·9 | — | 1·3 |
| 87 | 54 | Post | Av. | 22·6 | — | 2·8 |

*Abbreviations:* Av.—average grade malignancy.
o.c.—patient known to be taking an oral contraceptive.

0–22·6 × $10^{-15}$ with a mean of 3·7 × $10^{-15}$. Of the 36 samples examined, 8 gave a zero result and one was borderline.

The dissociation constant ($K_d$) observed for the oestradiol-receptor complex at 30° C. ranged from 1–5 × $10^{-10}$ M. This variation may be partly due to the error involved in determining the slope of the Scatchard plot, but the apparent

TABLE II.—*Clinical Data and Oestradiol Receptor Concentrations in Benign Breast Biopsies*

| Case No. | Age of patient | Menopausal status | Histology | Oestradiol receptor concn. × $10^{15}$ (moles/mg. tissue) | $K_d$ × $10^{10}$ (M) |
|---|---|---|---|---|---|
| 4 | 35 | Pre | BMD | 0 | — |
| 11 | 48 | Pre | Fibroadenoma | 0 | — |
| 18 | 46 | Pre | BMD | 0 | — |
| 19 | 64 | Post | BMD | 0 | — |
| 20 | 49 | Pre | BMD | 0 | — |
| 22 | 41 | Pre | Fibroadenoma | 0 | — |
| 24 | 36 | Pre | BMD | 0 | — |
| 25 | 52 | Post | BMD | 0 | — |
| 32 | 46 | Pre | BMD + Cyst | 0 | — |
| 34 | 51 | Pre | BMD | 0 | — |
| 36 | 55 | Post | Fat necrosis | 0 | — |
| 38 | 49 | Pre | BMD | 0 | — |
| 39 | 51 | Post | BMD | 0 | — |
| 40 | 39 | Pre | Fibroadenoma | 0 | — |
| 42 | 33 | Pre | Fibroadenoma | 0 | — |
| 45 | 43 | Pre | BMD + Cyst | 0 | — |
| 47 | 20 | Pre (o.c.) | BMD | 0 | — |
| 50 | 47 | Pre | BMD | 0 | — |
| 51 | 47 | Pre | BMD | 0 | — |
| 57 | 33 | Pre | Fibroadenoma | 0 | — |
| 60 | 25 | Pre | Fibroadenoma | 0 | — |
| 61 | 26 | Pre (o.c.) | Fibroadenoma | 0 | — |
| 63 | 43 | Pre | BMD | 0 | — |
| 64 | 46 | Pre | BMD | 0 | — |
| 68 | 45 | Pre | BMD | 0 | — |
| 69 | 37 | Pre | BMD | 0 | — |
| 71 | 40 | Pre | Fibroadenoma | 0 | — |
| 75 | 43 | Pre (o.c.) | BMD | 0 | — |
| 67 | 47 | Pre | BMD | 0 | — |
| 76 | 43 | Pre | BMD | 0 | — |
| 77 | 41 | Pre | BMD | 0 | — |
| 79 | 40 | Pre | BMD | 0 | — |
| 80 | 38 | Pre | Fat necrosis | 0 | — |
| 83 | 45 | Pre | BMD | 0 | — |
| 88 | 19 | Pre | Fibroadenoma | 0 | — |
| 95 | 47 | Pre | BMD | 0 | — |
| 96 | 60 | Post | BMD | 0 | — |
| 97 | 44 | Pre | BMD | 0 | — |
| 82 | 37 | Pre | Fibroadenoma | 0·3 | 4·5 |
| 91 | 39 | Pre | Fibroadenoma | 0·4 | 3·0 |
| 70 | 19 | Pre (o.c.) | Fibroadenoma | 0·6 | 2·1 |

*Abbreviations:* BMD—benign mammary dysplasia.
o.c.—Patient known to be taking an oral contraceptive.

value of the constant is also influenced by factors such as the concentration of endogenous oestradiol and the relative amount of low-affinity receptor present. Bearing in mind these limitations it is likely that the same type of receptor is involved in all cases.

The histological grading of each carcinoma is shown in Table I. The tumour was entirely intraduct in three biopsies, two of which were devoid of oestradiol binding sites, while the third case had a level of 1·05. The ten high grade carcinomas had oestrogen receptor levels between 0 and 8·3, the 29 average grade tumours between 0 and 22·6 and the 11 low grade tumours between 0 and 3·9.

A study of the cells composing each carcinoma showed three different types. First a small round cell reminiscent of the cell seen in salivary gland tumours and thought to have a myoepithelial origin. Secondly, a large adenomatous epithelial cell with appearances suggesting origin from the lining breast duct epithelium and thirdly a cell intermediate between the first two types of cell. Although a slightly greater proportion of the negative oestrogen-receptor carcinomas were composed of the first type of cell compared with the positive receptor carcinoma cases no significant correlation was found between the carcinoma cell type and oestrogen binding level.

No other histological feature was seen which might explain the variation in oestrogen binding levels of the biopsies invaded by carcinoma.

*Benign breast disease and normal tissue*

Of the 41 benign breast biopsies shown in Table II, evidence of specific oestradiol binding was found in only 3, and in these the receptor concentration was very low, the maximum value being $0{\cdot}6 \times 10^{-15}$ moles/mg. Thirty-six of the 41 patients were premenopausal, including the 3 which gave a positive result. Four of the patients were known to be taking an oral contraceptive, one of whom had a positive receptor level.

Histologically, this group consisted of 27 biopsies with features of benign mammary dysplasia, 2 with fat necrosis and 12 with fibroadenomata (Table II). The 3 positive cases with oestradiol receptor levels of 0·3, 0·4 and 0·6 were fibroadenomata, predominantly pericanalicular in type and in 2 of them myoepithelial cells surrounding the breast tubules were prominent and vacuolated, but overall they could not be distinguished histologically from the other 8 fibroadenomata.

The majority of biopsies with benign mammary dysplasia showed adenosis, fibrosis, duct ectasia and stagnation. Inflammatory cell infiltrates were usually scanty while apocrine metaplasia, small cysts and minor epitheliosis were present only in a minority of sections. Severe epitheliosis was not seen.

No oestrogen sites have been found in samples classified as normal or in areas of uninvaded tissue surrounding tumours.

### DISCUSSION

The results of this study confirm that a high proportion of human mammary carcinomas contain specific high-affinity oestradiol receptors which are not detected in normal breast tissue or in the majority of benign lesions. These results are consistent with the finding that some but not all breast cancers can concentrate oestrogens *in vivo* (Folca, Glascock and Irvine, 1961; Demetriou *et al.*, 1964; Desphande *et al.*, 1967; Braunsberg, Irvine and James, 1967; Pearlman *et al.*, 1966) and also with *in vitro* studies which have shown by various methods that some breast tumours contain specific oestrogen receptors similar to those found in the uterus (Jensen *et al.*, in press; Korenman and Dukes, 1970; Johansson, Terenius and Thoren, 1970).

The assay technique used in the present study has the advantages of being simple, quantitative and suitable for routine application. A result can be obtained on a single biopsy sample within a few hours, and 20 samples can easily be handled by one person in a day. Only 100 mg. of tissue is required per assay and the amount of isotope consumed is very small. The use of the charcoal incubation procedure to eliminate binding to non-specific low-affinity sites results in an improved accuracy and sensitivity. This may account for the proportion of positive results obtained being higher than in comparable studies using different techniques. It is uncertain whether a zero result represents a complete absence of oestradiol receptors or simply a concentration below the limits of sensitivity of the assay (approx. $0 \cdot 1 \times 10^{-15}$ moles/mg.). However, in most of these samples no oestradiol was bound whatsoever, although in a few cases there was slight residual binding of up to 1 % of the labelled oestradiol to low-affinity receptors.

In considering the variability of oestrogen receptor concentration in the malignant tumours, the effect of endogenous hormone must be taken into account. The assay method measures only vacant receptor sites. Those sites already occupied by endogenous hormone are largely excluded because the slow rate of dissociation of the complex permits only a small proportion of them to equilibrate under the conditions employed. In order to measure the total number of sites, it would therefore be necessary to determine the amount of endogenous oestrogen bound in each tumour supernatant. Although this was attempted, there was insufficient material available in most cases for accurate estimation. Thus in tumours which contained significant levels of endogenous oestrogen the result obtained may represent an underestimate of the true oestrogen receptor capacity. This could partly account for the fact that the measured receptor capacities of tumours from premenopausal women were on average lower than those of post-menopausal women. However, it has been found that in rat uterine supernatants the fraction of the total receptor sites occupied by endogenous oestradiol never exceeds 10 % of the total (Feherty *et al.*, 1970). If a similar situation exists in breast tumours the effect of endogenous hormone would be negligible.

No histological feature was found which might explain the variation in levels of oestradiol receptor in the carcinomas. Neither the proportion of tumour to surrounding connective tissue, nor the grading of the tumour according to the W.H.O. Classification of Breast Tumours correlated with the varying oestrogen binding levels. The 3 intraduct carcinomas had negative or low levels but some purely invasive carcinomas also had negative binding sites. Similarly there was no correlation with the extent of invasion by the carcinoma or the cell type composing the tumour.

Thirty-eight of the 41 patients considered histologically to have benign conditions of the breast had negative oestrogen binding site estimations. The 3 positive cases showed benign fibroadenomata which were predominantly pericanalicular in type. In 2 of these tumours the myoepithelial cells were vacuolated and prominent but there was no suspicion of a malignant change and there was no histological feature to distinguish them from the 8 other fibroadenomata. The 2 cases with fat necrosis of the breast showed no remarkable histological features. The remaining 28 benign biopsies all with negative oestrogen binding sites showed the complete range of histological appearances associated with benign mammary dysplasia although no marked epitheliosis was seen.

No obvious explanation has been found for the absence of oestrogen receptors in the majority of benign biopsies. Although the cellularity of these samples was generally lower than in the carcinomas, the difference was not sufficient to account for the failure to detect any receptors. However, the results are consistent with those obtained by Johannsson, Terenius and Thoren (1970) who found significant oestradiol binding in only 2 out of 26 benign tumours.

All the malignant biopsies studied were from primary tumours, except for case No. 62 which appeared to be a recurrence of a carcinoma in the other breast which was removed by mastectomy 7 years previously. Treatment in all cases consisted of mastectomy frequently followed by a course of radiotherapy depending on the extent of lymph node involvement. As the duration of this survey was less than 2 years, information on follow-up of the patients is very incomplete. One patient with advanced disease (No. 17) died shortly after operation and another (No. 23) has developed metastases in the region of the mastectomy scar and in the spine but as yet none of the others have shown any sign of recurrence. None of the patients has received steroid therapy. An evaluation of the potential clinical usefulness of the assay is, therefore, not possible from the results of this survey at present. However, the results of Jensen *et al.* (in press) indicate a positive correlation between a high concentration of oestrogen receptors and a favourable response to endocrine therapy. Confirmation of these findings will only be obtained by a long-term study of a very large number of patients.

This work was supported by the Cancer Research Campaign. The interest and support of Professor A. C. Thackray and Professor R. H. S. Thompson in this project is gratefully acknowledged, and also the technical assistance of Miss M. Ruzkova and Mr. A. Korda.

### REFERENCES

Braunsberg, H., Irvine, W. T. and James, V. H. T.—(1967) *Br. J. Cancer*, **21**, 714.

Brecher, P. I., Chabaud, J.-P., Colucci, V., DeSombre, E. R., Flesher, J. W., Gupta, G. N., Hughes, A., Hurst, D. J., Ikeda, M., Jacobson, H. I., Jensen, E. V., Jungblut, P. W., Kawashima, T., Kyser, K. A., Neumann, H.-G., Numata, M., Puca, G. A., Saha, N., Smith, S. and Suzuki, T.—(1971) in 'Advances in the Biosciences 7: Schering Workshop on Steroid Hormone "Receptors"'. Edited by G. Raspe. Braunschweig (Pergamon Press, Vieweg), p. 75.

Burton, K.—(1956) *Biochem. J.*, **62**, 315.

Demetriou, K., Crowley, L. G., Kushinsky, S., Donovan, A. J., Kotin, P. and MacDonald, I.—(1964) *Cancer Res.*, **24**, 926.

Desphande, N., Jensen, V., Bulbrook, R. D., Berne, T. and Ellis, F.—(1967) *Steroids*, **10**, 219.

Feherty, P. and Kellie, A. E.—(1971) in 'Some Implications of Steroid Hormones in Cancer'. Edited by D. C. Williams and M. H. Briggs. London (William Heinemann Medical Books), p. 49.

Feherty, P., Robertson, D. M., Waynforth, H. B. and Kellie, A. E.—(1970) *Biochem. J.*, **120**, 837.

Folca, P. J., Glascock, R. F. and Irvine, W. T.—(1961) *Lancet*, ii, 796.

Gorski, J., Toft, D., Shyamala, G., Smith, D., and Notides, A.—(1968) *Recent Prog. Horm. Res.*, **24**, 45.

Jensen, E. V., Block, G. E., Smith, S., Kyser, K. and DeSombre, E. R. in 'Estrogen, Target Tissue and Neoplasia'. Edited by T. L. Dao. Chicago (Chicago University Press) (in press).

JENSEN, E. V., DESOMBRE, E. R. AND JUNGBLUT, P. W.—(1967) in 'Endogenous Factors Affecting Host-tumor Balance'. Edited by T. L. Dao and S. Wood, Jr. Chicago (Chicago University Press), p. 15.
JENSEN, E. V., SUZUKI, T., KAWASHIMA, T., STUMPF, W. E., JUNGBLUT, P. W. AND DESOMBRE, E. R.—(1968) *Proc. natn. Acad. Sci., U.S.A.*, **59**, 632.
JOHANSSON, H., TERENIUS, L. AND THOREN, L.—(1970) *Cancer Res.*, **30**, 692.
KING, R. J. B., SMITH, J. A. AND STEGGLES, A. W.—(1970) *Steroidologia*, **1**, 73.
KORENMAN, S. G.—(1968) *J. clin. Endocr. Metab.*, **28**, 127.
KORENMAN, S. G., AND DUKES, B. A.—(1970) *J. clin. Endocr. Metab.*, **30**, 639.
MESTER, J., ROBERTSON, D. M., FEHERTY, P. A. AND KELLIE, A. E.—(1970) *Biochem. J.*, **120**, 831.
MOBBS, B. G.—(1966) *J. Endocr.*, **36**, 409.
PEARLMAN, W. H., DEHERTOGH, R., LAUMAS, K. R., BRUEGGEMANN, J. A. AND PEARLMAN, M. R. J.—(1966) in 'Steroid Dynamics'. Edited by G. Pincus, T. Nakao and J. F. Tait. New York (Academic Press), p. 159.
SANDER, S.—(1968) *Acta. path. microbiol. scand.*, **74**, 301.
SANDER, S. AND ATTRAMADAL, A.—(1968) *Acta path. microbiol. scand.*, **74**, 169.
SCARFF, R. W. AND TORLONI, H.—(1968) 'Histological Typing of Breast Tumours'. Geneva (World Health Organization).
SCATCHARD, G.—(1949) *Ann. N.Y. Acad. Sci.*, **51**, 660.